# Meaning of park-golf experience among older adults with physical disabilities: A Salutogenic approach

**DOI:** 10.1093/geroni/igaf122.2327

**Published:** 2025-12-31

**Authors:** Rokbit Lee, Sua Im, Yaeeun Yoo, Jinmoo Heo

**Affiliations:** Yonsei University, Seoul, Republic of Korea; Yonsei University, Seoul, Republic of Korea; Yonsei University, Seoul, Republic of Korea; Yonsei University, Seoul, Republic of Korea

## Abstract

Park-golf is a simplified and casual version of golf that has been popular among older adults in Asian countries. Park-golf has been appealing to older adults due to its shallow learning curve. This phenomenological study explored the meaning of park-golf experience using Salutogenesis Theory. Salutogenesis is a concept that focuses on origins of health, and it focuses on the resources that contributes to promoting health. The participants in this study were older park-golf players with physical disabilities in South Korea. A total of eight older adults were selected through snowball sampling who were members of local park-golf clubs and involved in playing park golf at least 5 years (mean age = 66.1). Semi-structured in-depth interviews were conducted with the participants. Analysis of the transcripts revealed the following three main themes: comprehensibility, manageability, and meaningful activity. The result demonstrated that Park-golf plays an important role in enhancing the comprehensibility of older adults’ lives, leading to increased predictability in life. Participants perceived that they had the ability and resources to cope with difficult moments in park golf, which contributed to effectively managing challenges in their daily lives. Participants have established their identity as accomplished park-golf players, and actively engage in social activities, contributing to meaningful and active aging within their local communities. This study emphasizes park-golf as a valuable leisure activity for older adults with physical disabilities, helping them manage daily stress, maintain a positive mindset, and build resilience for a meaningful life.

